# Molecular Alterations in Lung Adenocarcinoma With Ground-Glass Nodules: A Systematic Review and Meta-Analysis

**DOI:** 10.3389/fonc.2021.724692

**Published:** 2021-09-13

**Authors:** Zihan Wei, Ziyang Wang, Yuntao Nie, Kai Zhang, Haifeng Shen, Xin Wang, Manqi Wu, Fan Yang, Kezhong Chen

**Affiliations:** ^1^Department of Thoracic Surgery, Peking University People’s Hospital, Beijing, China; ^2^Health Science Center, Peking University, Beijing, China

**Keywords:** ground-glass-opacity, lung cancer, systematic review, molecular alteration, EGFR, PD-L1

## Abstract

**Background and Aims:**

Nodular ground-glass lesions have become increasingly common with the increased use of computed tomography (CT), while the genomic features of ground-glass opacities (GGOs) remain unclear. This study aims to comprehensively investigate the molecular alterations of GGOs and their correlation with radiological progression.

**Methods:**

Studies from PubMed, Embase, Cochrane Library, and Web of Science, using PCR, targeted panel sequencing, whole exosome sequencing, and immunohistochemistry, and reporting genomic alterations or PD-L1 expressions in lung nodules presenting as GGOs until January 21, 2021 were included in this study. Chi-square test, random-effects model, and Z-test analysis were adopted to analyze the data.

**Results:**

A total of 22 studies describing mutations in lung adenocarcinoma (LUAD) with GGOs were analyzed. EGFR was the most frequently mutative gene (51%, 95%CI 47%–56%), followed by TP53 (18%, 95%CI 6%–31%), HER2 (10%, 95%CI 0%–21%), ROS1 (6%, 95%CI 0%–18%), and KRAS (6%, 95%CI 3%–9%). The correlation between the frequency of EGFR mutation and radiological was observed and the differences were found to be not statistically significant in the subgroups, which are listed as below: radiological: gGGO 47.40%, 95%CI [38.48%; 56.40%]; sGGO 51.94%, 95%CI [45.15%; 58.69%]. The differences of the frequency of KRAS mutation in the different subgroups were also consistent with this conclusion, which are listed as: radiological gGGO 3.42, 95%CI [1.35%; 6.13%]; sGGO 12.27%, 95%CI [3.89%; 23.96%]. The pooled estimated rate of PD-L1 was 8.82%, 95%CI [5.20%–13.23%]. A total of 11.54% (3/26) of the SMGGNs were confirmed to be intrapulmonary spread by WES.

**Conclusions:**

Somatic genetic alterations are considered in early-stage GGO patients without distinct changes of the frequency following the progress of the tumor. This review sheds insight on molecular alterations in LUAD with GGOs.

## Introduction

Ground-glass opacities (GGOs), defined as hazy increased density of the lungs with bronchial and vascular margins on computed tomography (CT) (1, 2), often associate with lung cancers, especially lung adenocarcinomas (LUADs), and are commonly detected in East-Asia patients. GGOs, being radiologically distinct clinical entities, which were known to have an indolent clinical course, present a superior survival after resection, especially pure GGOs with a nearly 100% long-term disease-free survival (DFS), shown in many previous studies (3, 4), indicating the unique biology of GGOs. However, the molecular characteristics of GGO-associated lung cancers have not been systematically reviewed due to the limitation of sample size and different criteria used while reporting, and, therefore, the tumor evolutionary mechanism behind the slow-growing appearance in GGOs is not clear. In addition, there are many patients with synchronous multiple ground-glass nodules (SMGGNs) on their initial CT. And some of them are found to have an intrapulmonary spread, even if the initial lesions seem to be in a fairly early-stage.

Therefore, we meta-analyzed the extracted data under certain criteria to demonstrate the dynamic genomic alterations in the diversity of GGO patients. This review can provide a novel insight into the molecular alterations in LUAD patients with GGOs and new views for the biology behavior of GGOs.

## Methods

### Search Strategy

Three distinctive keywords were identified as follows: “ground-glass opacity”, “gene alterations”, and “PD-L1”. MeSH term database from the National Center for Biotechnology Information (NCBI) was searched to find all the possible expressions for these keywords which were defined as free words. The final search strategy was combined with both the MeSH terms and free words, which is listed as follows: #1: “GGO” OR “GGN” OR “ground glass opacity” OR “ground glass nodule” OR “ground glass nodules” OR “ground-glass opacity” OR “ground-glass nodule” OR “ground-glass nodules” OR “subsolid nodule” OR “subsolid nodules” OR “subsolid pulmonary nodules”, #2: “Gene” OR “Cistron” OR “Cistrons” OR “Genetic Materials” OR “Genetic Material” OR “genetic feature” OR “genetic characteristics” OR “genetic characteristic” OR “genetic features” OR “Genomic alteration” OR “Genomic alterations” OR “EGFR” OR “epidermal growth factor receptor” OR “TTF-1” OR “thyroid transcription factor 1” OR “ALK” OR “anaplastic lymphoma kinase” OR “KRAS” OR “Kirsten rat sarcoma” OR “HER2” OR “human epidermal growth factor receptor type 2” OR “oncogenic driver”, and #3: “PD-L1” OR “programmed cell death 1 ligand 1 protein” OR “PDL1” OR “CD274” OR “B7-H1” OR “B7H1”. “#1 AND #2” and “#1 AND #3” were searched in the four databases up to January 21, 2021, without language limitations, respectively.

### Selection Criteria

Firstly, all the papers retrieved from the search were screened by reviewing the titles and abstracts, during which period, reviews, case studies, editorials, meeting abstracts, and papers not meeting any of our search criteria were excluded. Then, the full contents of the rest papers were evaluated carefully to distinguish the ones that perfectly fit our inclusion criteria, analyzing the molecular alterations in a consecutive cohort of patients with GGOs, during which period, some papers were excluded for the following reasons (1): the cohort was developed to analyze the characters of the nodules with specific molecular alterations (2); insufficient data for analyses; and (3) papers not written in English. Two authors (ZWe and ZWa) conducted the procedure independently to evaluate the study eligibility for our review. This analysis was performed according to the Preferred Reporting Items for Systematic Reviews and Meta-Analyses (PRISMA) statement (5).

### Data Extraction

The following basic data were extracted from the selected papers: author(s), year of publication, size and region of the cohort, characteristics of the patients in the study, radiological and pathological details of the nodules, the methodological details, and relevant statistical findings for the entire cohort and/or by population subgroups. Two authors (ZWe and ZWa) collected these data independently, and any discrepancies between the two authors were resolved by discussions with a third author (KC).

### Statistical Analysis

We firstly performed a descriptive analysis summarizing all the rates of gene alterations reported in the eligible works. Then, the rates of the gene alterations which had been reported in more than three studies were pooled using random-effects meta-analysis models allowing for the inherent heterogeneity of observational studies (6), after a data-transformation and normality-check using the variance-stabilizing double-arcsine transformation method (7). Q and I^2^ statistics were calculated to assess the heterogeneity between study-specific estimates (8). Forest plots were adopted to show a graphical presentation of the meta-analysis results, whereas Z-test was applied to check the level of significance of the differences of the pooled estimated rates from different groups, where the values of p less than 0.05 were considered to be significant. The publication biases were assessed by Egger test, which is based on a weighted linear regression of the effect on its standard error. All the analyses were implemented with R (version 4.0.3).

### Quality of Evidence

In this systematic review, the 25 included studies were all cross-sectional studies. All the patients had been diagnosed with lung cancer before or during their treatment. The authors finalized the list of included articles through discussion and agreement. Data from the articles were independently extracted by two authors (ZWe and ZWa) who were not involved in any of the reviewed studies. As recommended by the Agency for Healthcare Research and Quality, the assessment of the methodological quality of the included studies was made from 11 perspectives with the Cross-Sectional/Prevalence Study Quality, a scoring system specific for a cross-sectional study (**Supplementary Table 1**).

## Results

### Study Selection

**Figure 1:**
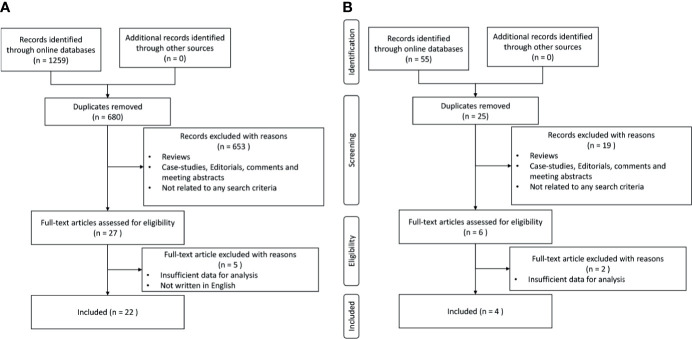
Process of study selection. **(A)** Study selection with genetic alterations. **(B)** Study selection with PD-L1 expressions.

After removing the duplicated records, 680 records related to gene alterations and 25 records related to PD-L1 expression were selected for further assessment with the titles and abstracts. From the remaining records, 27 records related to gene alterations and 6 records related to PD-L1 expression were carefully selected through the evaluation of the full contents as the second round of selection. Finally, 22 gene-alteration-reported articles and 4 PD-L1-expression-reported articles were included in the following analysis, as shown in a PRISMA diagram (**Figure 1**).

### Study Characteristics

All the cohorts from the 25 studies included were composed of Asians (**Table 1**), except that one cohort (29) also included Caucasians. In the 22 cohorts reporting gene alterations, the median cohort size was 135 [interquartile range (IQR): 25–210], of which, 16 cohorts included patients with solitary pulmonary nodules, while 6 cohorts put their attention on multiple pulmonary nodules, and the pathological subtypes of all these nodules were adenocarcinoma. While nearly half of the studies (11/22) focused on early-stage LUAD, 8 of the 22 studies included some stage III/IV cases (3 articles did not mention the clinical stage of the nodules). No subgroup analysis was performed due to the lack of data. Only two studies used whole exosome sequencing in their analysis, other than PCR or targeted gene sequencing. Among the four articles reporting PD-L1 expression included (**Table 1**) in our review, only one article reported gene alterations at the same time. All four cohorts were formed with Asians, two with Chinese, and two with Japanese. No significant publication bias were seen in the analysis (EGFR, p = 0.9419; KRAS, p = 0.7106; ALK, p = 0.0918; PD-L1, p = 0.89).

**Table 1:** Characters of the studies included in the meta-analysis.

Study ID	Region	Cohort size	Method_gene		Genes tested	EGFR mutation rate
Zhao et al. (9)	Chinese	529	qPCR & Immunohistochemical		EGFR、KRAS、HER2、ALK、BRAF、RET、ROS1、PIK3CA、NRAS	54.82%
Aoki et al. (10)	Japanese	25	PCR		EGFR、KRAS	40.00%
Dai et al. (11)	Chinese	204	qPCR		EGFR	53.43%
Suda et al. (12)	Japanese	1871	unclear		EGFR	50.61%
Min et al. (13)	Chinese	338	direct dideoxynucleotide sequencing		EGFR	64.79%
Zou et al. (14)	Chinese	209	PCR		EGFR	73.68%
Sugano et al. (15)	Japanese	59	non-radioactive single-strand conformation polymorphism		EGFR、KRAS	49.15%
Liu et al. (16)	Chinese	78	qPCR		EGFR	33.33%
Yang et al. (17)	Chinese	158	qPCR		EGFR、KRAS、ALK	62.66%
Lu et al. (18)	Chinese	156	qPCR		EGFR	48.08%
Chung et al. (19)	Korean	24	nested PCR		EGFR	41.07%
Hsu et al. (20)	Chinese	67	PCR		EGFR	55.22%
Ko et al. (21)	Korean	215	PCR		EGFR、ALK	54.63%
Tomita et al. (22)	Japanese	68	PCR		EGFR	72.06%
Chen et al. (23)	Chinese	39	DNA sequencing		EGFR、KRAS、BRAF、PIK3CA、TP53、ALK、ROS1、RET	56.52%
Kobayashi et al. (24)	Japanese	96	reverse transcriptase-PCR		EGFR、KRAS、ALK、HER2	64.42%
Li et al. (25)	Chinese	120	WES*			50.00%
Ren et al. (26)	Chinese	31	PCR & WES*			17.39%
Wang et al. (27)	Chinese	212	PCR		EGFR、KRAS	36.79%
Usuda et al. (28)	Japanese	56	Cycleave PCR		EGFR	67.86%
Lui et al. (29)	Asian & Caucasian	224	unclear		EGFR、KRAS	32.59%
Hong et al. (30)	Korean	116	PCR		EGFR	56.03%
**Study ID**	**Region**	**Cohort size**	**Antibody**		**Cut-off value**	**PD-L1 expression**
Wu et al. (31)	Chinese	233	E1L3N		5%	14.16%
Suda et al. (32)	Japanese	45	E1L3N		1%	4.44%
Toyokawa et al. (33)	Japanese	189	SP142		5%	9.52%
Zhao et al., 2018 (9)	Chinese	328	28-8, SP142, E1L3N, BP6001		5%	6.40%

*Whole exosome sequencing or next generation sequencing was used in the research lots of alterations reported, so the genes tested were omitted.

PCR, polymerase chain reaction; qPCR, quantitative polymerase chain reaction; NGS, next generation sequencing; WES, whole-exosome sequencing.

### Meta-Analysis

**Figure 2:**
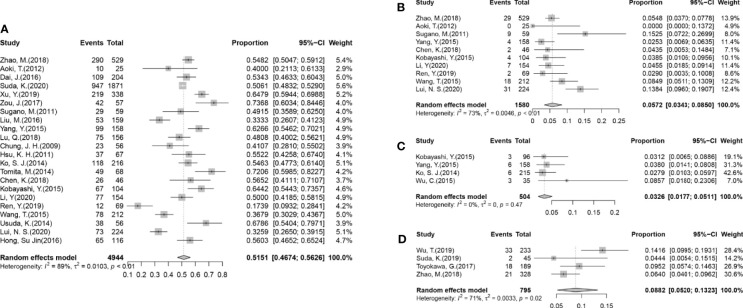
Forest plots from the meta-analysis of published gene alterations in GGOs. **(A)** The pooled estimated rate of the EGFR mutation in 22 articles. **(B)** The pooled estimated rate of the KRAS mutation in 10 articles. **(C)** The pooled estimated rate of the ALK rearrangement in four articles. **(D)** The pooled estimated rate of the PD-L1 expression in four articles.

Being the most validated genetic mutation, EGFR was the most prominent variation as well [51%, 95%CI (47%, 56%)], followed by TP53 [18%, 95%CI (6%, 31%)], HER2 [10%, 95%CI (0%, 21%)], ROS1 [6%, 95%CI (0%, 18%)], KRAS [6%, 95%CI (3%, 9%)] (**Figure 2**). Meanwhile, we summarized the rates of the top two validated gene alterations, EGFR mutation and KRAS mutation, to conduct a subgroup analysis.

#### EGFR Mutation

All the 25 articles that were included reported the rates of EGFR mutations in their cohorts (**Table 1**), in which 2,536/4,944 cases (51.29%) were found to harbor EGFR mutations. After performing a meta-analysis with the random-effects model (**Figure 2A**), the pooled estimated rate of EGFR mutations was found to be 51.51% [95%CI (46.74%, 56.26%)].

**Figure 3:**
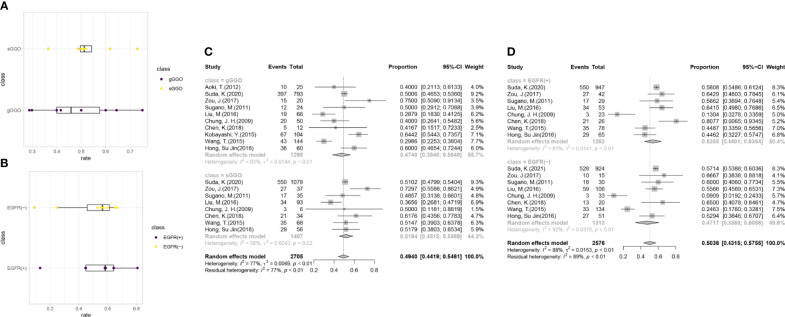
Subgroup analysis for the EGFR mutations. **(A)** The distribution of the EGFR gene mutation rates in the subgroups with a different radiological density. **(B)** The distribution of sGGOs in the subgroups with a different status of EGFR mutation. **(C)** Forest plots from the subgroup meta-analysis of EGFR alterations in GGOs with different radiological subtypes. **(D)** Forest plots from the subgroup meta-analysis of the percentage of sGGOs in GGOs with a different status of EGFR mutation. gGGO, ground-glass dominant GGO; 50%<G/T ratio ≤ 100%; sGGO, solid dominant GGO; 0<G/T ratio ≤ 50%.

Further analyses were conducted according to the radiological subgroups with the random-effects model (**Figure 3**). A G/T ratio, defined as the ratio of the ground-glass opacity (GGO) component to the tumor size at CT, ≥50% is suggested to be a sign of pathologically noninvasiveness. Additionally, the rates of lymph node metastasis range from 21% to 26% in lesions ≤3 cm with a G/T ratio ≤ 50% (34–36). Therefore, G/T ratio was used to divide the nodules into two groups in our review: gGGO (ground-glass dominant GGO) 50%<G/T ratio ≤ 100%; sGGO (solid dominant GGO) 0<G/T ratio ≤ 50%. The data of each subgroup were extracted from 10 articles that reported the necessary details according to the division criteria, and the EGFR mutation rate of each subgroup after analyzing with the random-effects model was listed in **Table 2**. It was found that the EGFR mutation rate has a marginal increment with the radiological progression of GGOs, but the difference was not statistically significant (p = 0.4828). Our results showed that with the radiological progression of GGOs, the frequency of EGFR mutation was stable.

**Table 2:** Details of the radiological subgroup analysis for the EGFR mutation and KRAS mutation by the random effect model.

EGFR	G/T ratio*	mut/total (n = 10)	Pooled estimated	95%CI	Heterogeneity		*Z-value^#^*	P-value*^#^*
					I^2^	P-value		
	gGGO	624/1,298 (n = 10)	47.40%	38.48%–56.40%	83%	<0.01	0.7018	0.4828
	sGGO	716/1,407 (n = 8)	51.94%	45.15%–58.69%	58%	0.02
	EGFR mut	sGGO/gGGO (n = 8)	pooled estimated	95%CI	Heterogeneity		*Z-value^#^*	P-value*^#^*
					I^2^	P-value
	EGFR (+)	716/1,263 (n = 8)	53.85%	44.01%–63.54%	81%	<0.01	0.6554	0.5122
	EGFR (–)	691/1,313 (n = 8)	47.17%	33.68%–60.86%	92%	<0.01
KRAS	G/T ratio*	mut/total (n = 5)	pooled estimated	95%CI	Heterogeneity		*Z-value^#^*	P-value*^#^*
					I^2^	P-value
	gGGO	14/309 (n = 5)	3.42%	1.35%–6.13%	0%	0.50	1.8075	0.0707
	sGGO	19/137 (n = 3)	12.27%	3.89%–23.96%	66%	0.05

*G/T ratio: gGGO, ground-glass dominant GGO; 0<G/T ratio ≤ 0.5; sGGO, solid dominant GGO; 0.5<G/T ratio ≤ 1.

^#^Z-value was calculated by Z-test, p < 0.05 is considered statistically significant.

Moreover, the mutation rates of EGFR subtypes were collected and analyzed (**Supplementary Figure 2**), and we found that the rate of L858R mutation was approximately equal to that of 19del mutation, composed over half of all EGFR mutations together. Though the rate of T790M mutation was relatively low (0.58%), there were still early-stage GGOs harboring T790M mutations.

In order to uncover whether EGFR mutation would have an influence on tumor progression, we divided the researches collected in our analysis into two groups by the mutation status of EGFR, among which, two studies included only cases with gGGOs and were excluded in the following analysis. The result shows that the proportion of the sGGOs was fairly the same in the groups with a different EGFR-mutation status, which is 53.85% [95%CI (44.01%, 63.54%)] in the EGFR(+) group and 47.17% [95%CI (33.68%, 60.86%)] in the EGFR (–) group, with a p-value at 0.5122 (**Table 2**). Though the heterogeneity between studies was still high, the result in each study can still confirm our results—sGGOs compose about 50% in whether EGFR(+) or EGFR (–) groups in each study.

#### KRAS Mutation

A total of 10 articles had reported the rates of KRAS mutation (**Figure 2B** and **Supplementary Figure 1**), in which 106/1,580 (6.71%) cases were reported to harbor the KRAS mutation, which was far less than the EGFR mutation. The pooled estimated rate of the KRAS mutation was 5.72% [95%CI (3.43%, 8.50%)]. Subgroups were also divided according to the criteria mentioned before. In the radiological subgroups, it was clear to demonstrate that the rate of the KRAS mutation increased with the decrease of G/T ratio numerically (gGGO 3.42%, sGGO 12.27%), but the difference was not statistically significant (p = 0.07), due to the large 95% confidence intervals and high heterogeneities (**Table 2**).

#### ALK Rearrangement

The rate of ALK rearrangement in GGOs, reported in 4 of the 22 studies, was 18/504 (3.57%) after enumeration (**Figure 2C**), and the pooled estimated rate was 3.26% [95%CI (1.17%, 5.11%)]. The heterogeneity of the studies reporting an ALK arrangement was fairly low (I^2^ = 0%, p = 0.47).

#### PD-L1 Expression

Among all 795 cases, 74 (9.31%) were found with the PD-L1 expression (**Figure 2D**). Though with a significant heterogeneity in the method and cut-off values assessing the PD-L1 expression, it can be confirmed that the rate of the PD-L1 expression in GGOs is fairly low, as the pooled estimated rate of the PD-L1 expression of the nodules was 8.82% (95% CI 5.20%–13.23%). There was only one article (33) that provided details in the subgroups, so a meta-analysis could not be performed on the subgroups. Still, it was shown in the articles that the rate of PD-L1 expression was significantly lower in GGOs (4.44%–14.16%) than solid nodules (18.36%–35.04%) in the same cohorts (9, 31–33).

#### Molecular Alterations in Synchronous Multiple Ground-Glass Nodules

A total of 6/22 articles included synchronous multiple ground-glass nodules (SMGGNs) in their cohorts, while only the data from 4 articles could be extracted for analysis. In the 123 cases with SMGGNs included, 8 cases (6.50%) possessed identical mutations in their resected nodules which were doubted to be an intrapulmonary spread. Only one article used whole-exosome sequencing (WES) and confirmed 3/26 (11.54%) cases to have an intrapulmonary spread. The genetic alterations of the cases are listed below (**Table 3**). The distribution of genetic alterations appeared to have no significant differences between genetic alteration rates in whether multicentric origin nodules (**Table 3A**) or intrapulmonary spread nodules (**Table 3B**).

**Table 3:** Gene mutations in synchronous multiple ground-glass nodules (SMGGNs).**TABLE 3A** Data extracted from four articles which showed the gene alterations among multicentric SMGGNs.

Author (year)	Gene_targeted	Amount	Leision_1	Leision_2	Leision_3	Leision_4	Leision_5
Liu, M (16).	EGFR	10	EGFR 19del	wild^#^			
		10	EGFR L858R	wild			
		8	EGFR L858R	EGFR 19del			
		1	EGFR 19del	wild			
		1	EGFR 19del	EGFR L858R			
		1	EGFR 19del	EGFR G719S			
		1	EGFR S768I	wild			
		1	EGFR L858R	EGFR 19del			
		1	EGFR L861Q	wild			
		1	EGFR L858R	EGFR L858R/19del			
Chung, J. H (19).	EGFR、KRAS	9	EGFR 19del	wild			
		6	wild	wild			
		4	EGFR 19del	EGFR L858R			
		1	EGFR 19del/KRAS	wild			
		1	EGFR 19del/F712L	wild			
		1	EGFR L858R	wild			
		1	EGFR L858R	G724S/L861Q			
		1	KRAS	wild			
Chen, K (23).	EGFR、KRAS、BRAF、TP53、ALK、ROS1、RET	1	EGFR others/TP53	wild			
		1	EGFR L858R	BRAF			
		1	EGFR L858R	wild			
		1	EGFR L858R/19del	wild			
		1	KRAS	wild			
		1	ROS1	wild			
Li, Y (25).	EGFR、KRAS、BRAF、TP53、ALK*	4	others	others			
		3	KRAS	others			
		3	others	others	others		
		2	EGFR	others			
		2	EGFR	EGFR			
		2	EGFR/TP53	EGFR			
		1	EGFR/TP53	others			
		1	EGFR/TP53	ALK			
		1	EGFR	ROS1			
		1	others	others	others	others	others
		1	EGFR	EGFR	others		
		1	EGFR	EGFR	EGFR		
		1	EGFR/TP53	others	others		

*whole exosome sequencing was used and the data showed only some specific gene alterations.

^#^wild means no target gene alteration was found in the research.

**Table 3B:** Three cases confirmed by Li, Y (25). which applied WES to analyze gene alterations in their research.

Author(year)	Method	Gene	Gene_alterations
Li, Y (25).	WES	patient_1	EGFR/others
		patient_2	EGFR/TP53/others
		patient_3	EGFR/TP53/others

## Discussion

This systematic review investigated the molecular alterations in lung nodules presenting as ground-glass opacities and analyzed the trend of tumor genetic alterations along with the radiological progress.

As it is known to us that EGFR mutation was first reported in 2004 (37), and associate with non-smokers, female, LUAD tightly (38, 39). EGFR mutations were present in 10% of cases in Caucasians, while 30% in East Asians (40, 41), which may explain why the cohorts included in this review were mostly Asian. Among all the reported genetic alterations analyzed in this review, EGFR mutation was clearly the most validated and highest incidence genetic alteration, which is similar to previous studies (41). Some researchers had mentioned in their study (42) that EGFR functioned in tumor genesis, and also played an important role in tumor processing (41, 43). To the best of our best knowledge, this review is the first meta-analysis for the EGFR mutation rate especially in GGOs, which shows that with the progression in radiological, there is no significant difference in the rate of EGFR mutation (p ≥ 0.05), suggesting that EGFR mutation, as a driver mutation for lung cancer, count for tumorigenesis in a relatively early stage, and maintain consistency in the progression of tumors. In addition, though relatively low, there were still early-stage GGOs harboring T790M mutations, indicating that T790M might play a role in tumorigenesis, which is known to associate with chemotherapy resistance.

Transforming from gGGOs to sGGOs have always been considered as a sign of tumor progression and is widely used in daily clinical work. So, we defined such transformation as tumor progression in our meta-analysis. Researchers have a heated discussion about the factors distinguishing between the easy-to-progress GGOs and indolent GGOs for a long time with no consensus, which mainly lie in a large size, the presence of a solid portion, old age, gene alterations, and so on (44–46). Unfortunately, our findings indicated that EGFR mutation has a little impact on tumor progression. Statistically, there is no difference between the distribution of gGGOs and sGGOs whether in the EGFR mutation group or in the wild type group. Whether some other signaling pathways can and under what conditions they will regulate the progression of GGOs, and whether there were any signs besides genetic alterations such as genetic heterogeneity or chromosome instability require more studies to confirm, which may help us get a deeper understanding of the biological behavior of GGOs.

When taking KRAS mutation as one of the earliest discoveries of genetic alterations in lung cancers (41, 47) and reported as very important for tumor progression (48) into account, there seemed to bear discrepancy at different stages of tumor progression. Though not statistically different, the frequency of KRAS mutation seems to increase with the increasing G/T ratio, suggesting a relationship between KRAS mutation and tumor progression, resulting in a higher frequency in more progressed lung nodules presenting with GGOs.

Using antibodies targeting the PD-1 pathway is a promising and effective option of immunotherapy, a newly developed treatment of NSCLC (49, 50), where PD-L1 is used as a biomarker to predict the immunotherapy response (51). We clearly showed that the incidence of PD-L1 expression was much lower in GGOs than pure-solid tumors in several articles, which was verified by Suda et al. in a clinical experiment including 124 qualified patients (4% vs. 25%, p < 0.01) (32). Wu et al. also found in a small-size cohort that even for the same patient, the volume of synchronous GGOs showed no significant change before and after treatment (4,160.2 vs 4,185.5 mm^3^, p = 0.6050) than solid nodules (52). Therefore, it is predictable that PD-1 treatment is less effective for patients with GGOs.

When compared to lung cancer presenting as solid nodule(s), Zhao et al (9) reported that the EGFR mutation rate was higher in solid nodules than in GGOs, especially the subtype mutation of 19 del, agreeing with previous studies (14). They also reported that in patients with GGOs, there are significantly more frequent HER2 mutations (p = 0.033), while less frequent ALK translocations (p = 0.014) and PIK3CA mutations (p = 0.012), compared to patients with solid nodules (GGOs/Solid nodules = 529/718). However, contrary to other studies in our analysis, Hong et al. (30) find that EGFR mutations were significantly more frequent in tumors with GGOs than in solid tumors, which may be caused by the different cohort sizes, regions of the cohorts, and the methods used to detect the mutations, suggesting that more researches are needed to elucidate the difference in the rates of mutations between GGOs and solid nodules.

Despite PCR, WES, and WGS which are used in the studies included in our study, with the development of molecular diagnostic technology, the field of liquid biopsy has gradually developed a lot. Analyzing circulating tumor DNA (ctDNA) at the genomic level, a newly-emerged non-invasive approach, is proposed to have the ability to distinguish between malignant and benign disease (53, 54) and at the same time detect the molecular alterations carried by the nodules, and then guide targeted therapy and immunotherapy. However, it has been shown that indolent GGO-predominant lung cancers shed lower-level ctDNA, which is less detectable to help identify cancer in patients (55). Therefore, for very early lesions, such as GGOs, it is difficult to achieve an early diagnosis by ctDNA SNV testing under the current technology limitations with a low sensitivity.

Two studies reported independently that different pathological lesions could share identical mutations even in pre-invasive LUADs, such as AAH and AIS lesions (56, 57). This review also points out that different GGOs could share the same mutation in patients with SMGGNs, shows that SMGGNs might have an intrapulmonary spread, despite the multicentric regions. Detecting the mutation status of a specific gene by PCR is only a small fragment of the whole genome and does not represent the expression status of the whole genome, so using whole exon sequencing (WES) or whole-genome sequencing (WGS) to determine an intrapulmonary metastasis of GGO sounds more convincing. In the 26 cases with SMGGNs reported by Li et al. (25) using WES, 3 cases (11.54%) were confirmed to have an intrapulmonary spread. Despite the articles analyzed in our review, we noted that Li et al. (58) reported another two cases to be an intrapulmonary spread by WES in a case report. Though EGFR mutations were found in four of the five confirmed cases, it is still too early to come to a conclusion that specific molecular alterations are associated with the intrapulmonary spreading of GGOs. We need to be concerned that though GGO is usually considered an early-stage lesion, it has a certain probability of metastasis. However, the exact mechanism of metastasis in GGOs, non-invasive cancer, is still unclear. We noted that these GGOs were all in a close proximity which might result in dissemination along the airway. Furthermore, whether these multiple GGOs sharing the same mutation affects the prognosis needs to be explored by an in-depth longer follow-up clinical and mechanistic analysis.

With the development of an immune checkpoint inhibitor treatment, especially the inspiring results of neoadjuvant immune therapy, a series of studies have focused on the immune-environment of early-stage lung cancer patients. The TRACEx cohort reported that sparsely infiltrated tumors exhibited a waning of neoantigen editing during tumor evolution, compared with immune-infiltrated tumor regions exhibiting an ongoing immunoediting, with either loss of heterozygosity in human leukocyte antigens or depletion of expressed neoantigens (59).Recently, some researchers have used single-cell tumor sequencing to map the tumor microenvironment and have found that GGO has less endothelial cell angiogenesis, downregulated EGR1 expression, upregulated KLF6 expression, a significantly higher proportion of NK cells, and showing a marked metabolic disorder and immune response stress, compared to an advanced lung cancer (60, 61). These studies, although with a limited sample size, initially revealed distinct immune mechanisms in GGOs from non-GGO lung adenocarcinomas, helping us to further understand the essence of the inert progression of GGO and to identify the nodules with a poorer prognosis at an early stage.

## Conclusion

Our research revealed that EGFR mutation is not associated with the radiological progression of GGOs, which means EGFR mutation was a driver mutation for lung cancer in a fairly early stage, and maintains consistency in the progression of tumors. On the contrary, the frequency of KRAS mutation was higher in progressed lung nodules, indicating a position for KRAS mutation in tumor progression. Immunotherapy, as one of the recently discovered effective therapies for advanced lung cancer, is less effective against GGOs, which may be due to the low expression level of PD-L1 in early-stage lung cancer, found by our research. Though GGOs are usually considered early-stage lesions, there does have a possibility for SMGGNs to have an intrapulmonary spread, the mechanism behind which is still unclear. The limitation of our meta-analysis lies in its retrospective design; postsurgical follow-up or treatment plans at recurrence would differ among attending surgeons. Also, the high heterogeneity between the methodologies and results of researches is another limitation of our research. Overall, this review summarizes the published estimates of the rates of molecular alterations in lung nodules presenting as GGOs, which may help clinical treatment decisions for GGOs and provide a novel insight in revealing the molecular alterations behind GGOs.

## Data Availability Statement

The original contributions presented in the study are included in the article/**Supplementary Material**. Further inquiries can be directed to the corresponding author.

## Author Contributions

ZWe and ZWa conceived and planned the experiments. ZWe and ZWa carried out the experiments. ZWe, ZWa, HS, XW, and MW contributed to the data preparation. ZWe, ZWa, YN, and KZ verified the analytical methods. ZWe, ZWa, YN, KZ, HS, XW, and MW contributed to the interpretation of the results. ZWe and ZWa took the lead in writing the manuscript. All authors contributed to the article and approved the submitted version.

## Funding

This study was supported by the National Natural Science Funds (grant No. 82072566) and Peking University People’s Hospital Research and Development Funds (grant No. RS2019-01).

## Conflict of Interest

The authors declare that the research was conducted in the absence of any commercial or financial relationships that could be construed as a potential conflict of interest.

## Publisher’s Note

All claims expressed in this article are solely those of the authors and do not necessarily represent those of their affiliated organizations, or those of the publisher, the editors and the reviewers. Any product that may be evaluated in this article, or claim that may be made by its manufacturer, is not guaranteed or endorsed by the publisher.
