# Supplementary material for: Molecular Alterations in Lung Adenocarcinoma With Ground-Glass Nodules: A Systematic Review and Meta-Analysis
Source: Front Oncol. 2021 Sep 13;11:724692. doi: 10.3389/fonc.2021.724692 (PMC8475014; doi:10.3389/fonc.2021.724692)
Supplement: Supplementary file 1 [file DataSheet_1.docx]

Supplementary Material

# Supplementary Figures

**Supplementary figure 1: Subgroup analysis for the rate of KRAS mutations**


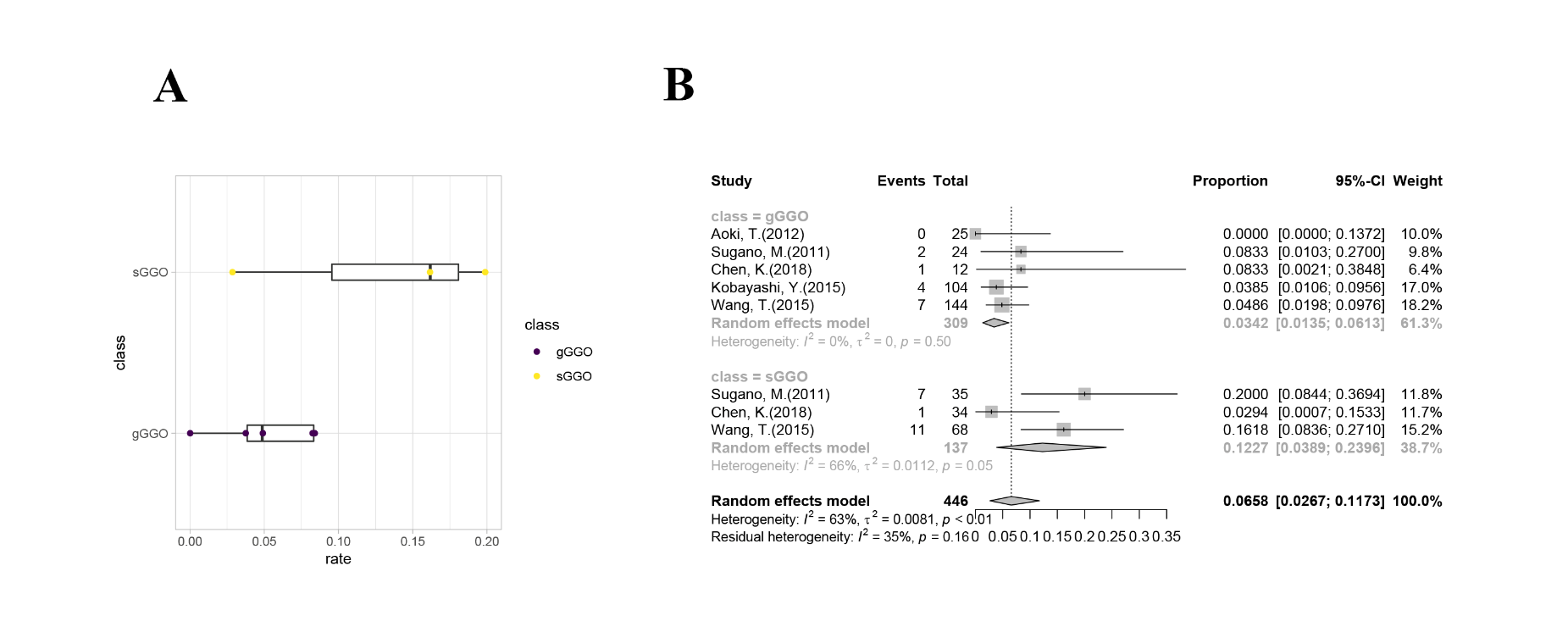


(A). The distribution of KRAS gene mutation rates in different radiological subgroups.

(B). Forest plots from subgroup meta-analysis of KRAS alterations in GGOs with different radiological subtypes.

gGGO: ground-glass dominant GGO, 50%<G/T ratio≤100%; sGGO: solid dominant GGO, 0<G/T ratio≤50%

Supplementary figure 2: Forest plots from meta-analysis of the rates of subtypes of EGFR mutation in GGOs


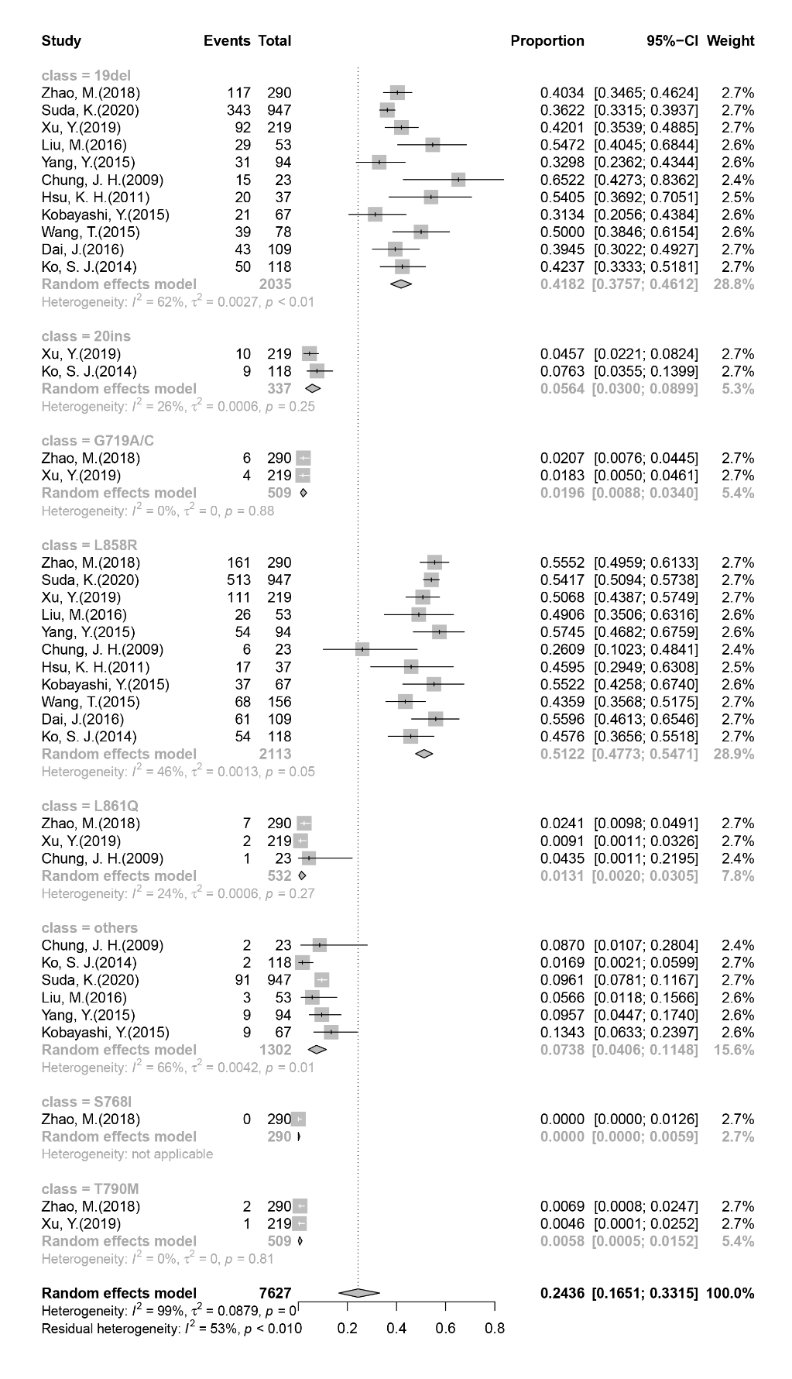


# Supplementary Tables

Supplement Table 1: Evaluating quality of evidence of all included articles.

| Source | A | B | C | D | E | F | G | H | I | J | K |
| --- | --- | --- | --- | --- | --- | --- | --- | --- | --- | --- | --- |
| Zhao, M.(2018) | * | * | * | * | UC | * | UA | - | UA | * | UA |
| Aoki, T.(2012) | * | * | * | * | UC | * | * | - | UA | * | * |
| Dai, J.(2016) | * | * | * | * | UC | * | * | - | UA | * | UA |
| Suda, K.(2020) | * | * | * | * | UC | * | * | - | UA | * | * |
| Xu, Y.(2019) | * | * | * | * | UC | * | * | - | UA | * | * |
| Zou, J.(2017) | * | * | * | * | UC | * | * | - | UA | * | UA |
| Sugano, M.(2011) | * | * | * | * | UC | * | UA | - | UA | * | UA |
| Liu, M.(2016) | * | * | * | * | UC | * | * | - | UA | * | * |
| Yang, Y.(2015) | * | * | * | * | UC | * | * | - | UA | * | UA |
| Lu, Q.(2018) | * | * | * | * | UC | * | * | - | UA | * | * |
| Chung, J. H.(2009) | * | * | * | * | UC | * | * | - | UA | * | UA |
| Hsu, K. H.(2011) | * | * | * | * | UC | * | * | - | UA | * | UA |
| Ko, S. J.(2014) | * | * | * | * | UC | * | * | - | UA | * | UA |
| Tomita, M.(2014) | * | * | * | * | UC | * | * | - | UA | * | UA |
| Chen, K.(2018) | * | * | * | * | UC | * | * | - | UA | * | * |
| Kobayashi, Y.(2015) | * | * | * | * | UC | * | * | - | UA | * | * |
| Li, Y(2020) | * | * | * | * | UC | * | * | - | UA | * | UA |
| Ren, Y.(2019) | * | * | * | * | UC | * | - | - | UA | * | UA |
| Wang, T.(2015) | * | * | * | * | UC | * | * | - | UA | * | UA |
| Usuda, K.(2014) | * | * | * | * | UC | * | * | - | UA | * | UA |
| Lui, N. S.(2020) | * | * | * | * | UC | * | * | - | UA | * | UA |
| Hong, Su Jin(2016) | * | * | * | * | UC | * | * | - | UA | * | UA |
| Wu, T.(2019) | * | * | * | * | UC | * | * | - | UA | * | UA |
| Suda, K.(2019) | * | * | * | * | UC | * | UA | - | UA | * | UA |
| Toyokawa, G.(2017) | * | * | * | * | UC | * | * | - | UA | * | UA |

A: Define the source of information (survey, record review); B: List inclusion and exclusion criteria for exposed and unexposed subjects (cases and controls) or refer to previous publications; C: Indicate time period used for identifying patients D: Indicate whether or not subjects were consecutive if not population-based; E: Indicate if evaluators of subjective components of study were masked to other aspects of the status of the participants; F: Describe any assessments undertaken for quality assurance purposes (e.g., test/retest of primary outcome measurements); G: Explain any patient exclusions from analysis; H: Describe how confounding was assessed and/or controlled; I: If applicable, explain how missing data were handled in the analysis; J: Summarize patient response rates and completeness of data collection; K: Clarify what follow-up, if any, was expected and the percentage of patients for which incomplete data or follow-up was obtained; * represents Yes; - represents No; UA means the item was unavailable to assess the article; UC represents unclear;
